# A Longitudinal Study of Financial Difficulties and Mental Health in a National Sample of British Undergraduate Students

**DOI:** 10.1007/s10597-016-0052-0

**Published:** 2016-07-29

**Authors:** Thomas Richardson, Peter Elliott, Ron Roberts, Megan Jansen

**Affiliations:** 1 Mental Health Recovery Team North, Solent NHS Trust, St. Mary’s Community Health Campus, Milton Road, Portsmouth, PO3 6AD UK; 2 School of Psychology, University of Southampton, Southampton, SO17 1BJ UK; 3 Psychology, Kingston University, London, UK; 4 Department of Psychology, Kingston University, Surrey, KT1 2EE UK

**Keywords:** Debt, Financial, Mental Health, Undergraduate, Student

## Abstract

Previous research has shown a relationship between financial difficulties and poor mental health in students, but most research is cross-sectional. To examine longitudinal relationships over time between financial variables and mental health in students. A national sample of 454 first year British undergraduate students completed measures of mental health and financial variables at up to four time points across a year. Cross-sectional relationships were found between poorer mental health and female gender, having a disability and non-white ethnicity. Greater financial difficulties predicted greater depression and stress cross-sectionally, and also predicted poorer anxiety, global mental health and alcohol dependence over time. Depression worsened over time for those who had considered abandoning studies or not coming to university for financial reasons, and there were effects for how students viewed their student loan. Anxiety and alcohol dependence also predicted worsening financial situation suggesting a bi-directional relationship. Financial difficulties appear to lead to poor mental health in students with the possibility of a vicious cycle occurring.

## Introduction

University represents a high risk time for mental health problems, with the start of university coinciding with the mean age of onset for many psychiatric disorders (Reavley et al. 2012). A United States (US) nationwide survey reported that almost half of all university-aged students have a psychiatric disorder which has functionally impaired them during the last academic year; however similar rates were reported for similar aged peers who did not attend university (Blanco et al. 2008). Similarly, Eisenberg et al. (2007) found that 15.6 % of US university students met criteria for a depressive or anxiety disorder. In Turkey, Bayram and Bilgel (2008) found moderately severe depression in 27 % of students and moderately severe anxiety in 47 %. Research also suggests that mental health may worsen over the course of university: Andrews and Wilding (2004) found that 9 % of United Kingdom (UK) students without a history of mental health problems at the start of university went on to develop clinical depression halfway through their degree. They also found that 20 % became clinically anxious over this time period. 

The potential impact of poor mental health amongst students has raised growing concerns with studies reporting it to interfere with university attendance, as well as reducing the likelihood of completing university (Blanco et al. 2008). High rates of substance use and alcohol use disorders are reported in students (Dawson et al. 2004; Slutske 2005), though rates may be similar to non-student populations (Blanco et al. 2008). Research in the US has also shown a high prevalence of suicidal ideation in students (Garlow et al. 2008).

One factor which has consistently been shown to predict poor mental health in students is financial difficulties. A number of studies examining UK based students have shown that mental health problems are linked to financial problems (Andrews and Wilding 2004; Roberts et al. 2000, 1999), level of debt (Carney et al. 2005) and concern about finances (Cooke et al. 2004; Jessop et al. 2005). The pooled findings from a meta-analysis by Richardson et al. (2013) found that 41.7 % of those with a mental health disorder report being in debt, in comparison to 17.5 % who report having no debt. For those who were in debt, 15.5 % had a mental health disorder in comparison to 8.9 % of those not in debt. A statistically significant relationship was also found between debt and depression, suicide completion or attempt, problem drinking, drug dependence, neurotic disorders and psychotic disorders (Richardson et al. 2013). The chief methodological issues identified with research in the area concern the use of non-validated measures of mental health problems, as well as the noted paucity of longitudinal studies.

Three longitudinal studies conducted with students are of particular relevance. Cooke et al. (2004) followed students for 3 years and found those with a high level of concern about their finances had a greater deterioration in mental health over time. Richardson et al. (2015a) examined the impact of the recent rise in tuition fees for UK students on mental health, finding no significant impact with those paying more having poorer mental health at only one out of four time points. However, using the same data Richardson et al. (2015b) found that financial difficulties in students increased eating disorder risk in students up to a year later. The relationship was partly bi-directional with eating disorder risk also increasing the risk of financial difficulties 3 months later. It may therefore be that it is financial difficulties such as ability to pay the bills which is more important than size of student loan.

At present therefore there has been little longitudinal research on the relationship between finances and mental health in students, and in particular only one previous study has examined whether finances predict poor mental health or vice versa in students. The present study therefore aimed to address this gaps in the literature by measuring a range of mental health symptoms and financial variables over time in a UK student sample.

## Methods

### Design

This study uses data from a prospective cohort study on tuition fees amount and mental health in students (Richardson et al. 2015a). This same data set has also been examined in relation to eating disorder risk and financial difficulties (Richardson et al. 2015b). In the current study a longitudinal design was used to assess whether financial variables influence changes in mental health overtime in undergraduate students.

### Measures

The following standardised measures were used to assess mental health. For all the measures higher scores represent more severe symptoms/worse mental health.

- Clinical Outcomes Routine Evaluation-General Population Version (CORE-GP) (Sinclair et al. 2005): This is a 14 item measure of global mental health with questions such as ‘I have felt optimistic about my future’ and ‘I have felt tense, nervous or unhappy’. In the current sample α at time 1 = .90.
- 7 Item Generalized Anxiety Disorder Questionnaire (GAD-7) (Spitzer et al. 2006): A seven item measure designed to screen for generalised anxiety disorder asking frequency of symptoms in past 2 weeks such as ‘trouble relaxing’ and ‘not being able to stop or control worrying’. Scores above 10 are suggestive of generalized anxiety disorder (Spitzer et al. 2006). This measure has also been used to measure anxiety the general population (Löwe et al. 2008). In the current sample α at time 1 = .91.
- Centre for Epidemiological Studies Depression Scale (CES-D) (Radloff 1977): This 20 item measure is designed to measure symptoms of depression in the past 2 weeks, and is designed specifically for epidemiological research with general population samples. Questions ask about frequency of symptoms such as ‘I was happy’ and ‘I felt that people disliked me’. Scores above 15 are suggestive of depression (Radloff 1977). In the current sample α at time 1 = .95.
- Perceived Stress Scale (PSS) (Cohen et al. 1983): This 10 questionnaire assess global perceived stress in the last month, using items such as how often individuals have felt ‘unable to control the important things in your life’ or ‘felt that things were going your way?’ In the current sample α at time 1 = .90.
- Alcohol Use Disorder Identification Test (AUDIT) (Saunders et al. 1993): This 10 item scale was developed to assess for alcohol problems via questions such as ‘How often do you have a drink containing alcohol?’ and ‘Have you or someone else been injured because of your drinking?’ Scores above 7 are suggestive of possible alcohol abuse or dependence (Babor et al. 2001). The AUDIT has been shown to be accurate in detecting alcohol problems in US university students (Kokotailo et al. 2004). In the current sample α at time 1 = .89.

The following measures of finances were used:

- Family Affluence Scale (FAS) (Currie et al. 1997): This four item measure is designed to measure the socio-economic status of adolescents via four questions such as ‘Does your family own a car, van or truck?’ This was used to measure the socio-economic status of student’s families. (α cannot be calculated for this measure as item responses differ between questions).
- Index of Financial Stress (IFS) (Siahpush and Carlin 2006): This measures financial difficulties/stress over the past 6 months via questions such as ‘Could not pay the mortgage or rent on time’. In the current sample α at time 1 = .71.
- Author constructed questions were developed on other financial variables. Participants were asked ‘*How stressed do you feel about your level of debt?*’ with response options ‘Not stressed’, ‘A Little stressed’, ‘Quite stressed’ or ‘Very stressed’. They were asked *‘Was this your first University choice?*’ with response options ‘Yes: was my first choice’, ‘No: Was an insurance or back-up choice’ or ‘No: I got the offer through clearing’. Participants were asked: ‘*How do you see your student loan?*’ with response options ‘Debt I will have to pay back’, ‘Debt I might have to pay back’ or ‘An extra tax (rather than debt)’. Finally, participants were asked ‘*Have you seriously considered abandoning your course because of any financial difficulties?’ (For example talking to your tutor about doing so, looking into career options etc.)*, with a Yes/No response. They were also asked *‘Did you seriously consider not coming to University due to financial concerns?’ (For example did you look into other career options, apply for jobs etc.)*, with a Yes/No response.

### Participants and Procedure

Recruitment is described in detail in the original paper (Richardson et al. 2015a, b). British first year undergraduate students were eligible to take part. Students were contacted through their university students union who were contacted by researchers and invited to advertise a survey examining factors which effect mental health in students. The universities included a wide range in terms of ranking and geographic location, and students were from a range of disciplines. 

The measures were completed online at four time points over the participants first 2 years at university. Each time point was 3–4 months apart, with the overall length from time 1 to time 4 being just over 1 year. Participants were only included in current analysis if they completed baseline and at least one other time point. A total of 454 participants were included with 38.1 % (n = 173) completing all four time points, 27.8 % (n = 155) completing three time points and 34.1 % (n = 155) completing two time points. The sample was 77.9 % (n = 352) female, 89.6 % (n = 405) white ethnicity, 5.7 % (n = 28) mixed ethnicity, 1.5 % (n = 7) Black, 1.5 % (n = 7) Asian, 1.1 % (n = 5) ‘Other’ and 0.8 % (n = 2) did not state. Ages ranged from 17 to 57 with a mean of 19.9 years. Eleven per cent (n = 50) reported being mature students (age 21 or over at start of university) and 8.8 % (n = 40) reported that they had a disability.

### Statistical Analyses

Missing data were filled in with the mode. All measures were normally distributed. Hierarchical, linear hierarchical multiple regression was used to see whether the financial variables (FAS, IFS, considering dropping out or not coming to university due to finances, whether got first choice, how stressed about debt) predicted scores at each time point. Demographic variables were also included in the model (age, gender, disability, mature student, ethnicity). For times 2 to 4 the baseline scores for that mental health measure were also included, for example to see if anxiety at time 3 was affected by demographics after accounting for anxiety score at baseline. The dummy variable was the most common variable, and listwise deletion was used for missing data in the regression. A linear regression was also used to see whether baseline mental health impacted follow-up IFS.

## Results

### Baseline Finances and Follow-Up Mental Health

The final linear regression models examining the impact on financial variables at baseline on follow-up mental health are shown in Tables 1 and 2. Female gender predicted higher anxiety and stress but lower alcohol dependence at baseline. Having a disability predicted poorer global mental health, higher depression, anxiety and stress at baseline and greater anxiety and stress at T2. Having a disability also predicted lower alcohol dependence at T4. Mature students had significantly lower alcohol dependence at baseline and time 2. There was no effect of age on any of the variables. 

**Table 1:** Final regression models of financial variables and follow-up anxiety, depression and stress

	GAD-7 (anxiety)				CES-D (depression)				PSS (stress)				
	Baseline	T2	T3	T4	Baseline	T2	T3	T4	Baseline	T2	T3	T4	
Overall model													
n	427	371	242	215	435	377	248	219	423	362	241	213	
F (df)	5.42*** (20, 406)	18.25*** (21, 349)	10.42*** (21, 220)	5.84*** (21, 193)	6.40*** (20, 414)	20.94*** (21, 355)	12.91*** (21, 226)	6.19*** (21, 197)	5.95*** (20, 402)	17.14*** (21, 340)	9.44*** (21, 219)		7.71*** (21, 191)
R^2^	.21	.52	.50	.39	.24	.55	.55	.40	.23	.51	.48		.46
Individual predictors (*β*)^a^													
Gender (female)	−.09*	−.07	−.07	−0.4	−.03	−.01	−.07	.02	−.11*	−.06	−.10		.02
(No disability) vs, disability	.14**	.08*	−.01	−.02	.14**	.05	−.03	−.03	.11*	.09*	.05		.02
(Not mature student) vs. mature student	−.08	−.01	.03	−.06	−.12	.03	−.06	−.01	−.12	.07	−.01		−.03
Age													
(17–19) vs. 20–29	.03	−.03	−.04	.06	.04	−.03	−.01	.05	.01	−.07	−.04		.05
(17–19) vs. 30+	−.02	.02	−.02	.02	−.04	−.04	.04	.05	.00	−.04	−.01		−.02
Ethnicity													
(White) vs. other	−.04	.06	−.02	.05	−.06	.05	.02	.03	−.08	.04	.01		.10
(White) vs. mixed	.02	.01	−.04	−.06	.05	.04	−.01	−.02	.02	.02	.03		.00
(White) vs. Asian	.03	−.01	−.05	−.06	−.03	.00	−.03	−.05	−.01	−.02	−.06		−.08
(White) vs. Black	−.03	.01	−.04	−.05	−.00	.02	−.05	−.03	.03	.03	−.04		−.06
Family affluence scale	−.03	−.03	.02	.00	−.02	−.00	−.02	−.02	−.03	−.07	−.05		−.05
Baseline index financial stress	.15*	.13*	.01	.05	.19**	.08	.00	.01	.17**	.06	.08		.14
How stressed about debt													
(Not at all) vs. a little	.06	.04	.05	.15*	.14**	.02	.04	.09	.13*	.10*	.09		.15*
(Not at all) vs. quite	.14**	.05	.04	.16*	.16**	.04	−.02	.12	.16**	.09	.03		.17*
(Not at all) vs. very	.09	−.04	−.02	.03	.11	−.03	−.07	.02	.17**	.00	−.06		.02
Considered abandoning studies due to finances													
(No) vs. yes	.11	.04	.10	.16	.12*	.09	.13*	.18*	.11	.01	.12		.13
Considered not coming to University due to finances													
(No) vs. yes	.12*	−.03	.01	−.06	.12*	.02	.13*	−.11	.06	.06	.03		−.03
How see student loan													
(Debt have to pay back) vs. an extra tax	−.09	.05	.03	.03*	−.04*	.06	.03	−.01*	−.04	.06	.06		.05
(Debt have to pay back) vs. debt might have to pay back	.01	.07	.04	.08	.07*	.05	.06	−.00	.10*	.03	.02		.03
University choice													
(First choice) vs. clearing	.05	.06	.03	.02	.07	.05	−.02	.08	.07	.03	−.02		.04
(First choice) vs. back-up	−.03	0.2	−.06	−.01	.00	−.03	−.10*	−.01	−.02	−.03	−.06		−.08
Baseline mental health measure score		.62***	.62***	.48***		.64***	.63***	.52***		.59***	.55***		.46***

*p < .05, **p < .01, ***p < .001

^a^Dummy/reference variables are in brackets. If *β* values are + then the comparison variable is associated with a higher score, if *β* values are −, then the dummy variable is associated with a higher score

**Table 2:** Final regression models of financial variables and later global mental health and alcohol dependence

	GORE-GP (global mental health)				AUDIT (alcohol dependence)			
	Baseline	T2	T3	T4	Baseline	T2	T3	T4
Overall model								
n	439	393	263	226	435	392	259	230
F (df)	7.47*** (20, 418)	23.62*** (21, 371)	10.91*** (21, 241)	6.82*** (21, 204)	2.46*** (20, 414)	55.36*** (21, 370)	43.17*** (21, 237)	16.86*** (21, 208)
R^2^	.26	.57	.49	.41	.11	.76	.79	.63
Individual predictors (*β*)^a^								
Gender (female)	−.04	.02	−.04	.03	.12*	.03	−.02	.05
(No disability) vs, disability	.14**	.05	.06	−.03	−.05	−.04	−.04	−.10*
(Not mature student) vs. mature student	−.06	.01	−.08	−.02	.19**	−.09*	−.03	−.09
Age								
(17–19) vs. 20–29	.06	−.08	−.01	.02	.07	.04	.01	.05
(17–19) vs. 30+	−.05	−.04	−.01	.00	.05	.03	−.00	.06
Ethnicity								
(White) vs. other	−.05	.07*	.02	.01	−.04	.03	−.00	.07
(White) vs. mixed	.04	.00	−.04	−.02	.08	−.00	.03	.05
(White) vs. Asian	−.01	−.03	−.05	−.06	−.13**	.00	−.01	−.02
(White) vs. Black	.00	.05	−.04	−.05	−.11*	−.02	.01	.01
Family affluence scale	−.08	−.02	−.06	−.07	.00	.01	.05	.01
Baseline index financial stress	.19**	.06	−.00	.00	.21**	.06	.09*	−.01
How stressed about debt								
(Not at all) vs. a little	.14**	.03	.09	.12	.08	−.06	−.08*	−.00
(Not at all) vs. quite	.18***	.04	.02	.15*	.00	.02	−.09*	−10
(Not at all) vs. very	.18**	.01	−.03	.01	−.04	−.07	−.10**	−.07
Considered abandoning studies due to finances								
(No) vs. yes	.09	.07	.06	.13	.08	−.02	.05	.08
Considered not coming to university due to finances								
(No) vs. yes	.10*	.04	.09	−.10	−.03	.06	.06	−.02
How see student loan								
(Debt have to pay back) vs. an extra tax	−.02	.08*	.04	−.01	.05	−.02	.00	.10
(Debt have to pay back) vs. debt might have to pay back	.04	.06	.09	.03	.05	.02	.03	−.01
University choice								
(First choice) vs. clearing	.05	.04	.03	.10	.04	0.01	.00	−.01
(First choice) vs. back-up	−.00	−.04	−.02	−.04	.01	−.06*	−.03	−.01
Baseline mental health measure score		.66***	.61***	.53***		.84***	.86***	.75***

*p < .05, **p < .01, ***p < .001

^a^Dummy/reference variables are in brackets. If *β* values are + then the comparison variable is associated with a higher score, if *β* values are −, then the dummy variable is associated with a higher score

Other ethnicity (compared to white) was associated with poorer global mental health at time 2, and white ethnicity was associated with greater alcohol dependence at baseline when compared to those of black or Asian ethnicity. Family affluence was not related to any variables. Greater financial stress predicted greater anxiety, depression, stress, alcohol dependence and poorer global mental health at baseline. Greater financial stress at baseline also predicted greater anxiety at T2 and greater alcohol dependence at T3. Greater subjective stress about debt predicted greater anxiety at baseline and T4, greater depression at baseline, greater stress at baseline T2 and T4, and poorer global mental health at baseline and T4. However, those who were less stressed about their finances had greater alcohol dependence at T4.

Considering abandoning studies due to financial reasons predicted higher depression at T3 and T4. Considering not coming to university for financial reasons predicted greater anxiety and poorer global mental health at baseline and greater depression at T3. Those who saw their student loan as debt they have to pay back had lower scores on anxiety at T4 than those who saw it as an extra tax. However, those who saw it as debt they had to pay back had more severe depression than those who saw it as an extra tax at baseline and T4. Those who saw it as debt they might have to pay back had more severe depression and stress at baseline than those who saw it as debt they have to pay back. Finally those who got their first choice university had more severe depression than those who got their back-up choice at T3, and more severe alcohol dependence at T2.

### Baseline Mental Health and Follow-Up Finances

A regression model with baseline IFS, FAS demographics and all of the mental health measures significantly predicted IFS at T2: *F*(14,357) = 52.5, *p* < .001, *R* ^*2*^ = .67. Individual significant predictors of higher IFS at T2 were baseline IFS (*β* = .71, *p* < .001) being age 30+ (compared to 17–19): *β* = .09, *p* < .01, Other ethnicity (compared to white): *β* = .08, *p* < .01 and low Family Affluence: *β* = −.08, *p* < .05. None of the mental health measures at baseline were significant predictors.

The model significantly predicted IFS at T3 *F*(14,233) = 21.7, *p* < .001, *R* ^*2*^ = .57. Individual significant predictors of higher IFS at T2 were baseline IFS (*β* = .76 *p* < .001), being age 30 (compared to 17–19): *β* = .15, *p* < .01, Other ethnicity (compared to white): *β* = .15, *p* < .001 and baseline CORE-GP score (*β* = .26, *p* < .05).

Finally, the model significantly predicted IFS at T4 *F*(14,204) = 11.7, *p* < .001, *R* ^*2*^ = .45. Individual significant predictors of higher IFS at T2 were baseline IFS (*β* = .57 *p* < .001) being Other ethnicity (compared to white): *β* = .16, *p* < .01 and baseline AUDIT score (*β* = .13, *p* < .05).

## Discussion

The present study examined the longitudinal relationship between financial variables and mental health in a UK student population using standardised measures. Greater financial stress such as being unable to pay the bills predicted poorer global mental health and higher anxiety, depression, stress and alcohol dependence when examined cross-sectionally. This corroborates findings from many previous studies (Lange and Byrd 1998; Roberts et al. 1999, 2000; Stuhldreher et al. 2007). The findings are at odds with Ross et al. (2006) who found that lower debts predicted poorer mental health, this may be due to the fact that their study focused on medical students specifically compared to the wider population in the current sample.

The majority of the previous literature was based on various author constructed questionnaires for financial and health measures (Richardson et al. 2013), which consequently can reduce the reliability and validity of these measures. As a result, the present study benefits from the use of standardised measures for both financial and mental health variables. An additional strength of this study is that the relationship between financial variables and mental health were investigated in a longitudinal manner. This is in contrast to the majority of the literature, which is predominately cross-sectional in nature and therefore limits the possibility of inferring causality (Richardson et al. 2013). The present study provides evidence in favour for financial stress predicting higher anxiety at 3–4 months and alcohol dependence at 6–8 months later after controlling for demographics and symptoms at baseline. This is in line with (Cooke et al. 2004) who in a 3 year study found that those with higher financial concern had a greater increase in symptoms over time.

Female gender also predicted higher anxiety and stress and lower alcohol dependence when examined cross-sectionally, which is in line with existing studies showing higher rates of anxiety disorders in women (Vesga-Lopez et al. 2008; Xu et al. 2012). Having a disability was also associated with having poorer global mental health, higher depression, anxiety and stress when examined cross-sectionally. Furthermore, having a disability appears to exacerbate anxiety and stress symptoms at T1, but lower alcohol dependency at T4. However, only 8.8 % of the students had a disability and this consequently may underestimate the size of the relationship. It is also possible that students reported mental health problems as a disability, thus by definition they are likely to score higher on measures of mental health difficulties.

Evidence has arisen which suggests that the relationship between finances and mental health may in actual fact be attributed to amount of stress about debt rather than actual debt (Lange and Byrd 1998; Selenko and Batinic 2011). The present findings suggest that greater stress about debt predicted greater anxiety, depression, stress and poorer global mental health when examined cross-sectionally. Greater subjective stress about debt exacerbated anxiety, depression, stress, as well as global mental health over time which corroborates previous literature’s findings (Cooke et al. 2004; Jessop et al. 2005). However, those who were less stressed about their financial debt had greater alcohol dependence at the T4. This could perhaps be attributed to the students feeling able to spend more money on alcohol, and therefore being more prone to develop problems. This is in line with Richardson et al. (2015a, b) who found that those paying lower tuition fees had more alcohol problems. The existing literature examining subjective stress about debt in comparison to debt itself is rather limited and requires further investigation, particularly in the student population. The findings of this study highlight the importance of stress and worry about debt, and it might be that psychological interventions that target cognitive biases and processes such as rumination may have a positive impact on mental health.

The present study found a mixed picture with regards to how students saw their loans: those students who regarded their student loan as debt rather than as an extra tax had lower scores on anxiety measures, but had higher scores on depression at T4. Additionally, those students who considered abandoning their studies due to financial reasons, as well as those who considered not coming to university for financial reasons predicted higher depression at T3. These findings emphasise the detrimental impact of financial difficulties on the academic performance of students.

The present study also suggests a possible bi-directional relationship with global mental health and alcohol dependence predicting higher levels of financial stress and vice versa. These findings are in line with previous work suggesting a ‘vicious cycle’ whereby poor mental health exacerbates financial difficulties and these financial difficulties then go on to effect mental health (Richardson et al. 2015b).

A number of limitations in this study need to be acknowledged. The findings should be interpreted with caution as several regressions were run so there is an increased risk of a type 1 error. In addition, the exact length of time between time points varied slightly. The results from the present study should be interpreted with caution as the self-selected sample, predominately female in nature, may not be representative of the general UK student population as a whole.

Future research is needed to confirm the possible bi-relationship between financial difficulties and mental health symptoms in students using a larger and more representative sample. It may also be beneficial with a larger sample to assess whether demographic variables influence the impact of financial variables on mental health. Richardson et al. (2015b) found that financial difficulties predicted eating disorder risk in female but not male students, but whether this is the case for other mental health problems such as depression and anxiety has yet to be seen. Finally the findings here on the importance of subjective stress about debt need to be examined more closely, looking at whether variables such as rumination, hopelessness and locus of control around finances mediates the impact of debt on mental health.

In conclusion, in students financial variables appear to lead to poor mental health rather than mental health problems leading to a deteriorating financial situation. However, there appears to be a bi-directional relationship between financial difficulties and global mental health and alcohol dependence, with finances worsening mental health and vice versa suggesting a vicious cycle developing. It is important for professionals working with students in a health or financial advice capacity to consider these findings.
